# Comparison of Lipase Production by *Enterococcus faecium* MTCC 5695 and *Pediococcus acidilactici* MTCC 11361 Using Fish Waste as Substrate: Optimization of Culture Conditions by Response Surface Methodology

**DOI:** 10.5402/2013/980562

**Published:** 2012-09-27

**Authors:** Vrinda Ramakrishnan, Louella Concepta Goveas, Bhaskar Narayan, Prakash M. Halami

**Affiliations:** ^1^Department of Food Microbiology, Central Food Technological Research Institute (CSIR), Mysore 570 020, India; ^2^Department of Meat, Fish & Poultry Technology, Central Food Technological Research Institute (CSIR), Mysore 570 020, India

## Abstract

A medium using fish waste as substrate was designed for production of lipase by *Enterococcus faecium* MTCC 5695 and *Pediococcus acidilactici* MTCC 11361. Medium components and culture conditions (fish waste protein hydrolysate (FWPH) concentration, fish waste oil (FWO) concentration, pH, temperature, and fermentation time) which affect lipase production were screened using factorial (5 factors *∗* 2 levels) design of which FWPH concentration, FWO concentration, and fermentation time showed significance (*P* < 0.05). The levels of these factors were optimized further by Box-Behnken design using response surface methodology (RSM). Optimized conditions were found to be 5% v/v FWO, 0.15 mg/mL FWPH and 24 h of fermentation time for MTCC 5695, and 4% v/v FWO, 0.15 mg/mL FWPH and 24 h of fermentation for MTCC 11361, which were further validated. Under optimized conditions, MTCC 5695 and MTCC 11361 showed 3.15- (543.63 to 1715 U/mL) and 2.3- (214.74 to 493 U/mL) fold increase in lipase production, respectively, as compared to unoptimized conditions.

## 1. Introduction

Lipases (triacylglycerol acylhydrolases EC 3.1.1.3) are a class of serine hydrolases which catalyze the hydrolysis of triglycerides to glycerol and free fatty acids over oil-water interface [1]. In addition, lipases catalyze the hydrolysis and transesterification of other esters as well as the synthesis of esters and exhibit enantioselective properties [2]. These unique properties of lipases make them a very important enzyme of industrial significance. Lipases are used in chemical processing, dairy industries for improvement of flavour, paper industries, oleochemical industries, pharmaceuticals, synthesis of surfactants, detergent industries, leather industries, and polymer synthesis [3, 4].

Lipases are produced by plants, animals, and microbes but only microbial lipases are found to be industrially important since they are diversified in their enzymatic properties and substrate specificity [5]. Bacterial lipases that are of commercial importance are obtained from *Achromobacter, Alcaligenes, Arthrobacter, Bacillus, Burkholderia, Chromobacterium*, and *Pseudomonas* [6, 7].

Lactic Acid Bacteria (LAB) are generally considered to be weakly lipolytic, as compared to other groups of microorganisms. The lipolytic activity by LAB plays an important role in the determination of special aroma of different cheeses [8, 9]. Since they are considered as generally recognised as safe (GRAS), they are used extensively as starter cultures in food and feed industries [10]. Although there are reports on lactic acid bacterial lipase production [11–13], they are fewer in comparison to other microorganisms like *Bacillus*.

Most research is now focused on the use of waste residues generated by industries as inexpensive substrates for microbial growth and metabolite production. Fish processing industries generate around 63.6 million metric tons (MMT) in which 2.8 MMT of waste are generated in India alone [14]. These wastes are a rich source of biomolecules such as lipids, proteins, chitin, collagen, minerals, and vitamins that can be recovered and utilized [15]. The lipids and proteins are extracted from the fish wastes either by addition of enzymes or by fermentation with LAB [16]. Lactic acid bacterial fermentation has been used for recovery of oil from fish viscera and also for extraction of proteins from shrimp waste and leather industry waste [15, 17, 18]. These lipidic carbon and nitrogen rich sources can be used as ample substrates for lipase production by LAB. However, these carbon and nitrogen supplements used must be optimized for maximal lipase production.

The most challenging task in optimization is the presence of interactive effects of media components and culture conditions. Response Surface Methodology (RSM) is a collection of statistical and mathematical techniques useful for developing, improving, and optimizing processes in which a response of interest is influenced by several variables and the objective is to optimize this response [19]. It defines the effect of independent variables, alone or in combination, on the processes and generates a mathematical model that describes the process [20].

In the present study, fish waste was used to design a medium for lipase production by *Enterococcus faecium *MTCC 5695 and *Pediococcus acidilactici *MTCC 11361. The significant parameters (media components and cultural conditions) on lipase production were identified using a factorial design and optimized using a Box-Behnken design. To the very best of our knowledge, there are no reports on the optimization of lipase production by LAB from fish waste by RSM.

## 2. Materials and Methods

### 2.1. Substrates and Chemicals

Fresh water fish visceral waste devoid of air bladder was collected from local fish markets in Mysore, India. *Enterococcus faecium* NCIM5335 (EF-35) used for extraction of fish oil was obtained from institute collection centre. All microbiological media were procured from Hi-Media (M/s Hi-Media, Mumbai, India). *Para*-nitrophenyl acetate (*p*-NPA) and *p*-nitrophenol were obtained from SRL (SRL chemicals, Bangalore, India). All other chemicals, solvents, and reagents used in the study were of analytical grade, unless otherwise mentioned.

### 2.2. Bacterial Strains and Inoculum Preparation

The lipase-producing strains used in the present study were isolated from fish processing waste. They were identified by 16S rDNA sequencing as *Enterococcus faecium *MTCC 5695 and *Pediococcus acidilactici *MTCC 11361 and deposited in National Culture Collection of Industrial Microorganisms, NCL, Pune. The strains were maintained as MRS glycerol stocks at −20°C and subcultured periodically. The gene sequences of *Enterococcus faecium *MTCC 5695 and *Pediococcus acidilactici *MTCC 11361 are deposited in NCBI with accession numbers HQ005360 and submission ID 1554294.

### 2.3. Extraction of FWO and FWPH from Fish Waste

Extraction of FWO was done as per the procedure detailed in Rai et al. [15] with slight modifications. Fresh water fish visceral waste devoid of air bladder was subjected to homogenization in a Waring blender (Stephen Mill, UM5 Universal, Hong Kong). The uniformly homogenized fish viscera was steam cooked at 85°C for 10 minutes to inactivate the inherent enzymes and microflora, followed by fermentation for 72 hours using EF-35. The fermented mass was then centrifuged at 6000 rpm for 20 min. FWO separated out into the top layer followed by protein rich residue layer. 

The protein hydrolysate was extracted from the protein rich residue layer as per Bhaskar et al. [21] with few modifications. The protein residue layer was extracted thrice with distilled water in the ratio 1 : 1 w/v. Protein extract obtained on centrifugation was subjected to lyophilisation to give FWPH, which was then dissolved in distilled water. The protein concentration was measured using Biuret's method [22].

### 2.4. Optimization Experiments

The medium used for optimization studies consisted of components as shown in Table 1.

#### 2.4.1. Screening of Significant Parameters Affecting Lipase Production by Factorial Design

The effect of pH (*X*1), temperature (*X*2, °C), time (*X*3, h), FWPH concentration (*X*4,  mg/mL), and FWO concentration (*X*5, %v/v) on lipase production by MTCC 5695 and MTCC 11361 was studied by a (5 factors ∗ 2 levels) factorial design encompassing 32 runs (Table 2). Lipase activity (*Y*) was determined as the response (dependent variable) and specifically designated as *Y*_*a*_ and *Y*_*b*_ for lipase activities of MTCC 5695 and MTCC 11361, respectively. FWPH and FWO were added as per amounts indicated in Table 2. The most significant factors influencing lipase production by MTCC 5695 and MTCC 11361 were chosen to enhance lipase production by Box-Behnken design.

#### 2.4.2. Box-Behnken Design

A Box-Behnken design for three factors encompassing 15 runs (Table 4) was applied for optimization of lipase production by MTCC 5695 and MTCC 11361. The factors namely FWO concentration (*X*1, %v/v), FWPH concentration (*X*2, mg/mL), and time (*X*3, h) were employed in three levels (−1, 0, + 1). Lipase activity (*Y*) was determined as the response (dependent variable). Lipase activities of MTCC 5695 and MTCC 11361 were designated as *Y*_1_ and *Y*_2_, respectively. FWPH and FWO were added as per amounts indicated in Table 4.

### 2.5. Lipase Assay

The optimization experiments were performed as presented in Tables 2 and 4 in 250 mL Erlenmeyer flasks containing 100 mL media. The experiments were performed in triplicates. The pH and temperature were maintained at 6.0 and 43°C (central values generated through factorial design), respectively. As per the time intervals indicated in Tables 2 and 4, sample aliquots were collected and centrifuged at 10,000 rpm for 10 min. Cell pellet was collected and sonicated in phosphate buffer (pH 7.0) for complete lysis. The lysed cells were centrifuged and lipase assay was performed for the cell free extract. 

Lipase activity was determined spectrophotometrically using *p*-NPA as the substrate as described by Wang et al. [23] with slight modifications. 300 *μ*L of cell supernatant and 900 *μ*L of acetonitrile : ethanol : phosphate buffer (pH 6.8) in ratio of 1 : 4 : 95 was mixed with 800 *μ*L of *p*-NPA (100 mM) in acetonitrile. This mixture was then incubated at 37°C for 15 minutes. The liberated *p-*nitrophenol was estimated at 408 nm. One unit of lipase activity is defined as the amount of enzyme required to liberate one *µ*mol of *p*-nitrophenol per minute under the standard assay conditions.

### 2.6. Statistical Analysis

The screening and optimization experiments were designed by STATISTICA software [24]. The data generated from the experiments were analyzed to obtain the optimized conditions by the same.

## 3. Results and Discussion

### 3.1. Selection of Substrate for Efficient Lipase Production by MTCC 5695 and MTCC 11361

Fish waste contains a rich source of both lipids and proteins and thereby can be applied as an efficient substrate for microbial growth and production of various metabolites [16, 25]. Henceforth, this study aims at the use of fish waste as an effective alternative for the carbon and nitrogen sources present in media currently used for cultivation of LAB. In this study, the carbon and nitrogen sources in the commercial MRS medium were replaced with FWO and FWPH, respectively, as indicated in Table 1. FWO and FWPH helped in enhanced lipase production by both the organisms thereby acting as a replacement for carbon and nitrogen sources, respectively. FWO consists mainly of triacylglycerols that comprises a variety of fatty acids that act as a remarkable lipidic carbon source for induction of lipase production [15]. On the other hand, FWPH serves as a rich source of proteins aiding in the luxurious growth of organisms and metabolite production. Moreover, most of the protein supplements used for the cultivation of LAB are of bovine origin which makes it unsuitable for use in food industries [26, 27].

### 3.2. Screening of Significant Independent Parameters by Factorial Design

The observed lipase activity values are shown in Table 2 along with the experimental runs. The influence of the chosen independent parameters on lipase production by MTCC 5695 and MTCC 11361 was studied by a (5 factors ∗ 2 levels) factorial design. ANOVA results for MTCC 5695 (Table 3(a)) and MTCC 11361 (Table 3(b)) indicate time (*X*3), FWPH concentration (*X*4), and FWO concentration (*X*5) to be the most significant independent parameters affecting lipase production (*P* ≤ 0.05). It was observed that time had a negative effect on lipase production, whereas FWO concentration and FWPH concentration had a positive effect. The production of lipase by MTCC 5695 (*Y*_1_) and MTCC 11361 (*Y*_2_) as a function of these parameters is represented by the following:

(1) Y1=−192.935+46.528∗X1+9.938∗X2− 6.19748∗X3+2909.623∗X4+114.0688∗X5,Y2=−58.3232+7.766719∗X1+0.844994∗X2−1.14688∗X3+437.0406∗X4+50.47033∗X5.

The optimum levels of the significant independent parameters were determined further by a Box-Behnken design and the insignificant independent parameters, that is, pH (*X*1) and temperature (*X*2) were maintained at the centre of their levels.

### 3.3. Optimization of Parameters for Lipase Production by Box-Behnken Design

The influence of FWO concentration (*X*1), FWPH concentration (*X*2), and time (*X*3) on lipase production was determined by Box-Behnken design as indicated in Table 4, which also presents the observed values for lipase activity of MTCC 5695 (*Y*_1_) and MTCC 11361 (*Y*_2_) at different combinations of the independent parameters. The lipase produced was found to vary from 437 U/mL to 1707 U/mL for MTCC 5695 and from 10.48 U/mL to 487.22 U/mL for MTCC 11361, in the fifteen experiments conducted which shows the strong influence of media components on the lipase production.

Tables 5(a) and 5(b) indicate the ANOVA table for MTCC 5695 and MTCC 11361, respectively.  Table 5(a) presents that among the independent variables, quadratic effect of FWO and FWPH showed significance on the response variable, whereas only linear effect of time had significance (*P* < 0.05). Moreover, the interactions between factors did not significantly influence (*P* ≥ 0.05) lipase production by MTCC 5695 (*Y*_1_) except for the interaction between FWO and time.  Table 5(b) depicts that among independent variables quadratic effect of FWO and FWPH showed significance on the response variable (*P* < 0.05), but both quadratic and linear effects of time did not show significance for lipase production by MTCC 11361 (*Y*_2_). Interactions between the independent variables did not show any significant effect (*P* ≥ 0.05). Similar studies stating the significant influence of sunflower oil and palm oil as inducible carbon sources on lipase production have been reported [28, 29].

**Figure 1:**
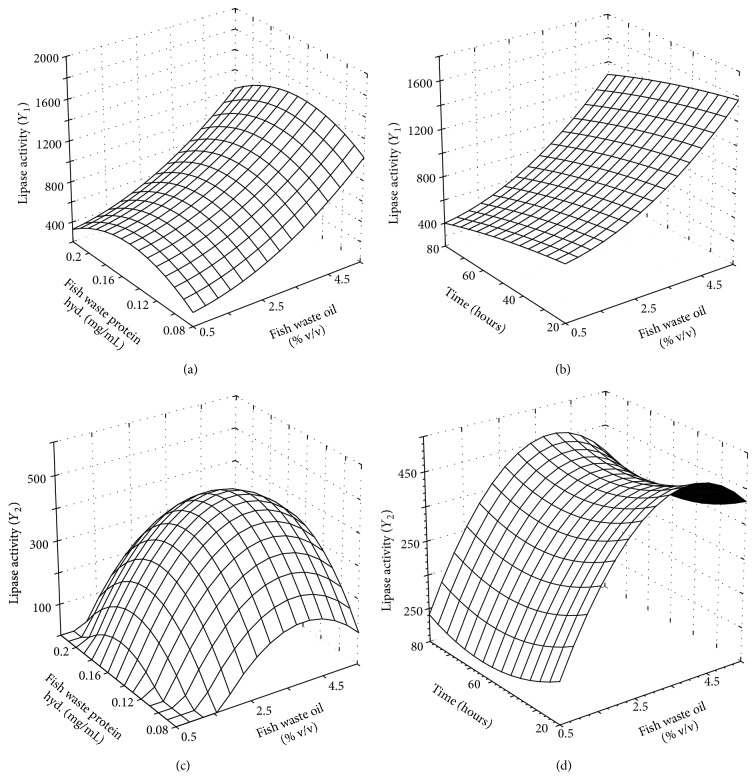
Three-dimensional plot showing the effect of: (a) FWPH concentration, FWO concentration; (b) FWO concentration, time; on lipase production by MTCC 5695 (c) FWPH concentration, FWO concentration; (d) FWO concentration, time; on lipase production by MTCC 11361. (FWPH: fish waste protein hydrolysates; FWO: fish waste oil).

The response surface graph for *Y*_1_ and *Y*_2_ as a function of FWPH concentration and FWO concentration is presented in Figures 1(a) and 1(c), respectively. It was observed that lipase production increased with increase in FWPH concentration up to 0.16 mg/mL beyond which there was a decrease in case of both the organisms probably due to inhibition of enzyme activity in the presence of excess nitrogen. A possible mechanism may be that FWPH is a complex nitrogen source and the cells may produce more protease for the degradation of FWPH before its utilization. This might result in lower production and higher degradation of the lipase [30]. The lipase production increased with increase in FWO concentration for MTCC 5695, whereas lipase production increased with increase in FWO concentration up to 4% v/v beyond which there was a decrease for MTCC 11361. The decrease in lipase production by MTCC 11361 beyond 4% v/v of FWO concentration may be due to the reason that high concentrations of FWO have more incidence of long chain fatty acids which might have an inhibitory effect on lipase production [5]. However, MTCC 5695 was found to be more tolerant to FWO. The influence of time and FWO concentration on *Y*_1_ and *Y*_2_ is presented in Figures 1(b) and 1(d), respectively. The figure clearly indicates that lipase production decreases with increase in time for MTCC 5695 however, a slight increase was observed after 48 h for MTCC 11361. This may be probably due to the fact that MTCC 5695 and MTCC 11361 achieve maximum growth in 24 h after which they enter the stationary phase resulting in a steady decline in lipase production.

**Figure 2:**
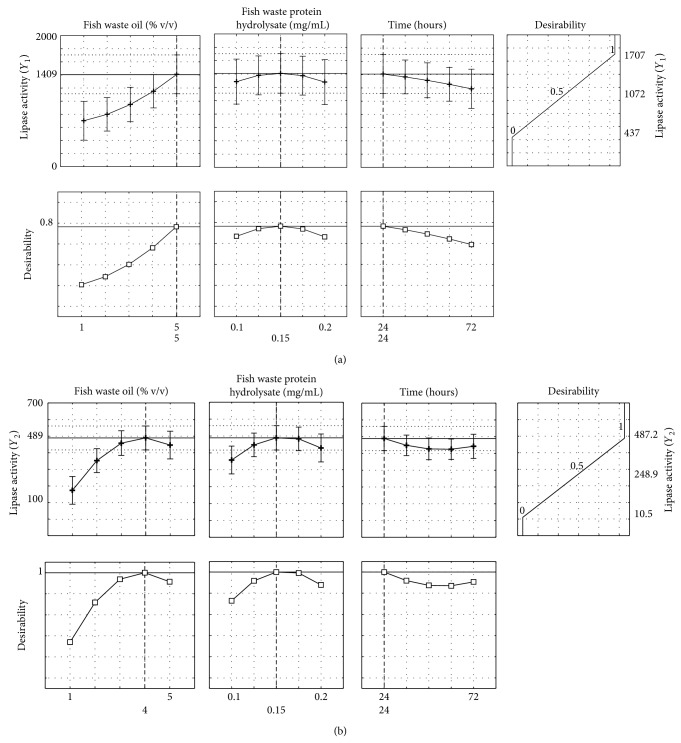
(a) Profiles for desirability levels of different factors (FWO—% v/v; FWPH—% v/v & time/hours) for optimum lipase activity by *Enterococcus faecium* MTCC 5695 (*Y*_1_); (b) Profiles for desirability levels of different factors (FWO—% v/v; FWPH—% v/v & time/hours) for optimum lipase activity by *Pediococcus acidilactici* MTCC 11361 (*Y*_2_).

The optimized levels of variables (*X*1, *X*2, and *X*3) were determined using desirability profiles for *Y*_1_ and *Y*_2_ (Figures 2(a) and 2(b)). The optimized factors for obtaining the highest *Y*_1_ were 5% v/v FWO concentration, 0.15 mg/mL FWPH concentration at 24 h of fermentation whereas for *Y*_2_, 4% v/v FWO concentration, 0.15 mg/mL FWPH concentration at 24 h of fermentation were found to be optimum. The response variables *Y*_1_ and *Y*_2_ were assigned a desirability of 1.0 for the highest observed value (*Y*_1_—1707 U/mL and *Y*_2_—487.22 U/mL) and a desirability of 0 for the lowest observed value (*Y*_1_—437 U/mL and *Y*_2_—10.48 U/mL) to get the overall desirability. The desirability function to get the optimum lipase production was fitted by the least square method. The level of variable giving the highest desirability (1.0) was selected as the optimum level.

The regression equation for lipase activity of MTCC 5695 and MTCC 11361, as a function of the three independent variables (*X*1, *X*2, and *X*3) and their linear and quadratic interactions, is represented by the following:

(2) Y1=−742.6+187.4∗X1+26.6∗X12+12560.0∗X2+50350.0∗X22+9.4∗X3+445.0∗X1∗X2−4.9∗X1∗X3+23.7∗X2∗X3,Y2=−708.7+276.0∗X1−37.0∗X12+12560.0∗X2−38200.6∗X22+0.1∗X32−11.6∗X3−2.4∗X1∗X2+0.3∗X1∗X3+21.5∗X2∗X3.

Coefficient of determination (*R* ^2^) is a measure of the strength of the linear relationship between the experimental and predicted values. *R* ^2^ for the correlation between the observed and predicted lipase activities of MTCC 5695 and MTCC 11361 was 0.9808 and 0.94246, respectively.

The model was validated by conducting experiments at 5% v/v FWO concentration, 0.15 mg/mL FWPH concentration at 24 h of fermentation for MTCC 5695 and 4% v/v FWO concentration, 0.15 mg/mL FWPH concentration at 24 h of fermentation for MTCC 11361. The experimental values of *Y*_1_ (1715 U/mL) and *Y*_2_ (493 U/mL) at these optimum conditions were quite close to the predicted values (1645.75 U/mL and 481.662 U/mL, resp.) which indicated that the model was highly significant. A fold increase of 3.15 and 2.3 was obtained, respectively, in lipase production for MTCC 5695 and MTCC 11361 by optimization using RSM (i.e. lipase activity of 543.63 U/mL and 214.74 U/mL under unoptimized conditions, resp.). This fold increase is more than the fold increase obtained by Sharma et al. [31] wherein a 1.6-fold increase in lipase production was observed in *Arthrobacter *sp. BGCC#490 and Kumari et al. [32] obtained 1.4-fold in lipase production in *Enterobacter aerogenes *under optimized conditions. Liu et al. [1] reported a 5-fold increase in lipase production by *Burkholderia* sp.

## 4. Conclusion

*Enterococcus faecium* MTCC 5695 and *Pediococcus acidilactici* MTCC 11361 were found to be potential lipase-producing strains using fish waste substrates. RSM was found to be an efficient methodology for rapid optimization of influencing parameters and development of polynomial equation for lipase production. The significance of this work is that it includes the use of an economical substrate for lipase production, which in turn diminishes the problem of waste disposal from fish processing industries. Moreover, the optimized conditions obtained from this study can be used for large-scale cost-effective production of lipase from LAB.

## Figures and Tables

**Table 1:** Composition of medium used for lipase production by MTCC 5695 and MTCC 11361.

Constituents	Amount (g/100 mL)
Magnesium sulphate	0.01
Manganese sulphate	0.005
Dipotassium hydrogen phosphate	0.2
Sodium acetate	0.5
Ammonium citrate	0.2
Tween 80	0.1
FWO∗	A^#^ (%v/v)
FWPH∗∗	B^#^ (mg/ml)

^*^As replacement for carbon source.

^**^As replacement for nitrogen source.

^#^Added as per concentrations indicated in Tables 2 and 4 (protein concentration of FWPH: 33.53 mg/mL as estimated by Biuret's method).

FWO: fish waste oil; oil recovered from fermented fish processing waste.

FWPH: fish waste protein hydrolysates; obtained after fermentation of fish processing waste.

**Table 2:** Factorial design for screening of significant independent variables affecting lipase production with the observed lipase activity values.

*X*1 pH	*X*2 temp (°C)	*X*3 time (h)	*X*4 FWPH (mg/mL)	*X*5 FWO (%v/v)	*Y*_*a*_ (U/mL)	*Y*_*b*_ (U/mL)
5	37	24	0.1	1	645	97.33
5	37	24	0.1	5	983	269.56
5	37	24	0.2	1	865.976	73.54
5	37	24	0.2	5	1428	391
5	37	72	0.1	1	243.98	41.2
5	37	72	0.1	5	1109.77	215.23
5	37	72	0.2	1	639	51.823
5	37	72	0.2	5	1208.67	287.45
5	47	24	0.1	1	908.743	158
5	47	24	0.1	5	1139.89	276
5	47	24	0.2	1	1097	89.779
5	47	24	0.2	5	1565.09	234
5	47	72	0.1	1	367.8188	68.9
5	47	72	0.1	5	890.098	213.856
5	47	72	0.2	1	737.51	49
5	47	72	0.2	5	1183	330
7	37	24	0.1	1	410	89.12
7	37	24	0.1	5	1430	274.33
7	37	24	0.2	1	1123	121
7	37	24	0.2	5	1603.45	409.66
7	37	72	0.1	1	378	34.567
7	37	72	0.1	5	967	208
7	37	72	0.2	1	809.09	58.526
7	37	72	0.2	5	1118	281
7	47	24	0.1	1	1010	91.23
7	47	24	0.1	5	1324	299
7	47	24	0.2	1	1238.45	145.89
7	47	24	0.2	5	1365.23	391.9
7	47	72	0.1	1	733	89.98
7	47	72	0.1	5	889	195
7	47	72	0.2	1	900.23	96
7	47	72	0.2	5	1203	310

*X*1: pH; *X*2: temperature, °C; *X*3: time, hours; *X*4: FWPH concentration (mg/mL); *X*5: FWO concentration, % v/v; *Y*_*a*_: lipase activity (U/mL) of MTCC 5695; *Y*_*b*_: lipase activity of MTCC 11361; FWPH: fish waste protein hydrolysates; FWO: fish waste oil.

**Table:** (a)

	SS	df	MS	*F*	*P* ^*^
Independent interactions					

pH	69276.12	1	69276.12	3.39826	0.077
TEMP	79015.43	1	79015.43	3.876011	0.059
TIME	707949.5	1	707949.5	34.72765	3.25*E* − 06
FWPH	677272.3	1	677272.3	33.22281	4.54*E* − 06
FWO	1665495	1	1665495	81.69894	1.66*E* − 09
Error	530029.8	26	20385.76		

Total SS	3729038	31			

^*^Values less than 0.05 indicate significance at 95% confidence interval.

**Table:** (b)

	SS	df	MS	*F*	*P* ^*^
Independent interactions					

pH	1930.3	1	1930.3	1.12393	0.299
TEMP	571.212	1	571.212	0.33259	0.569
TIME	24244.4	1	24244.4	14.1165	0.0008
FWPH	15280.4	1	15280.4	8.89709	0.0061
FWO	326049	1	326049	189.844	1.8*E* − 13
Error	44653.9	26	1717.46		

Total SS	412729	31			

^*^Values less than 0.05 indicate significance at 95% confidence interval.

**Table 4:** Actual levels of independent variables with the observed values of the response variable, Lipase activity (*Y*_1_ of MTCC 5695, *Y*_2_ of PA-63).

Run #	*X*1	*X*2	*X*3	*Y*_ 1_	*Y*_ 2_
1	1	0.1	48	598	10.48
2	5	0.1	48	1128	255.47
3	1	0.2	48	452	49.60
4	5	0.2	48	1160	293.61
5	1	0.15	24	437	214.96
6	5	0.15	24	1707	487.22
7	1	0.15	72	654	55.81
8	5	0.15	72	977	387.92
9	3	0.1	24	813	294.92
10	3	0.2	24	799	346.98
11	3	0.1	72	567	279.61
12	3	0.2	72	667	434.99
13	3	0.15	48	820	394.92
14	3	0.15	48	829	396.32
15	3	0.15	48	913	396.58

*X*1: FWO concentration, % v/v; *X*2: FWPH concentration, mg/mL; *X*3: time, hours.

*Y*_1_: lipase activity (U/mL) of MTCC 5695; *Y*_2_: lipase activity of MTCC 11361.

**Table:** (a)

	SS	Df	MS	*F*	*P* ^*^
Independent variables					

FWO (*L*)	1001820	1	1001820	177.0422	4.288*E* − 05
FWO (*Q*)	41780.83	1	41780.83	7.383533	0.0412
FWPH (*L*)	98	1	98	0.017319	0.9004
FWPH (*Q*)	58502.83	1	58502.83	10.33865	0.0236
TIME (*L*)	99235.12	1	99235.12	17.53689	0.0086
TIME (*Q*)	1020.519	1	1020.519	0.180347	0.6887

Interactions					

1∗2	7921	1	7921	1.399804	0.2899
1∗3	224202.3	1	224202.3	39.62116	0.0015
2∗3	3249	1	3249	0.574165	0.4827
Error	28293.25	5	5658.65		

Total SS	1474217	14			

^*^Values less than 0.05 indicate significance at 95% confidence interval.

**Table:** (b)

	SS	df	MS	*F*	*P* ^*^
Independent variables					

FWO (*L*)	149431.7	1	149431.7	42.7161	0.0012
FWO (*Q*)	81039.24	1	81039.24	23.16564	0.0048
FWPH (*L*)	10131.9	1	10131.9	2.896276	0.1495
FWPH (*Q*)	33675.83	1	33675.83	9.626472	0.0267
TIME (*L*)	4312.883	1	4312.883	1.232868	0.3173
TIME (*Q*)	5526.075	1	5526.075	1.579667	0.2643

Interactions					

1∗2	0.239121	1	0.239121	6.84E-05	0.9937
1∗3	895.5056	1	895.5056	0.255987	0.6344
2∗3	2668.756	1	2668.756	0.762883	0.4223
Error	17491.26	5	3498.252		

Total SS	303990.7	14			

^*^Values less than 0.05 indicate significance at 95% confidence interval.
